# A Low-Complexity ESPRIT-Based DOA Estimation Method for Co-Prime Linear Arrays

**DOI:** 10.3390/s16091367

**Published:** 2016-08-25

**Authors:** Fenggang Sun, Bin Gao, Lizhen Chen, Peng Lan

**Affiliations:** 1 College of Information Science and Engineering, Shandong Agricultural University, Tai’an 271018, China; sunfg@sdau.edu.cn (F.S.); lzchen@sdau.edu.cn (L.C.); 2 College of Communications Engineering, PLA University of Science and Technology, Nanjing 210007, China; feimaxiao123@gmail.com

**Keywords:** direction of arrival (DOA) estimation, co-prime array, ESPRIT, sparse array, equivalent DOAs

## Abstract

The problem of direction-of-arrival (DOA) estimation is investigated for co-prime array, where the co-prime array consists of two uniform sparse linear subarrays with extended inter-element spacing. For each sparse subarray, true DOAs are mapped into several equivalent angles impinging on the traditional uniform linear array with half-wavelength spacing. Then, by applying the estimation of signal parameters via rotational invariance technique (ESPRIT), the equivalent DOAs are estimated, and the candidate DOAs are recovered according to the relationship among equivalent and true DOAs. Finally, the true DOAs are estimated by combining the results of the two subarrays. The proposed method achieves a better complexity–performance tradeoff as compared to other existing methods.

## 1. Introduction

Direction of arrival (DOA) estimation is a crucial problem in various applications, such as radar, sonar, and wireless communications [1]. Various DOA estimation methods have been studied in uniform linear arrays (ULAs), including multiple signal classification (MUSIC) [2] and estimation of signal parameters via rotational invariance technique (ESPRIT) [3]. In [4], a Khatri-Rao product-based real-valued sparse estimation method is proposed. With respect to the random errors of sensor position, a stochastic framework is established to find the probability density function of the DOA-estimates [5]. However, most of the traditional DOA estimation schemes have focused on ULA structure [6], which, in fact, is not an optimal array geometry.

Recently, sparse array geometry has drawn lots of attention due to its high resolution [7,8,9]. An eigenstructure-based direction-finding algorithm is proposed in [7] for sparse uniform Cartesian arrays. In [8], an ESPRIT-based estimation method is proposed for a sparse array with two different sizes of spatial invariances. Moreover, the literature [9] introduces a novel direction-finding algorithm for a multiscale sensor array, which presents multiple scales of spatial invariance. Except for these sparse structures, the nested arrays [10] and co-prime arrays [11,12,13] have also attracted great attention. The nested array suffers from the mutual coupling problem due to some closely located sensors, while the co-prime array has less of a mutual coupling effect since sensor elements are sparsely located. Consequently, we consider the co-prime array structure in this paper. Various DOA estimation methods have been proposed for co-prime arrays [14,15,16]. A projection-like method is proposed in [14] to estimate DOAs by combining the results of the two subarrays. By applying the MUSIC algorithm, a total spectral search-based method (TSS) is proposed in [15], which, however, suffers from the high complexity caused by the spectral search step. By limiting the searching region to a small sector, a partial spectral search-based method (PSS) is proposed in [16]. It is shown in [16] that the PSS method can achieve almost the same estimation accuracy as TSS, but with a substantially reduced complexity. Further, the PSS method is extended to co-prime planar array structures to estimate two dimensional DOAs in [17]. However, since the complexity is mainly caused by spectral search, the works [16,17] still have a heavy computational burden.

To this end, in this paper, we propose a low-complexity DOA estimation method for co-prime linear arrays. For each subarray of the co-prime array, the true DOAs are mapped into their respective equivalent angles impinging on a traditional ULA with half-wavelength inter-element spacing, which can be the obtained by ESPRIT. Then, according to the relationship among true and equivalent DOAs, the candidate DOAs are recovered immediately. Finally, the true DOAs can be uniquely estimated by seeking the common angles recovered by the two subarrays. Simulation results are provided to verify the effectiveness of the proposed method.

## 2. System Model

**Figure 1:**
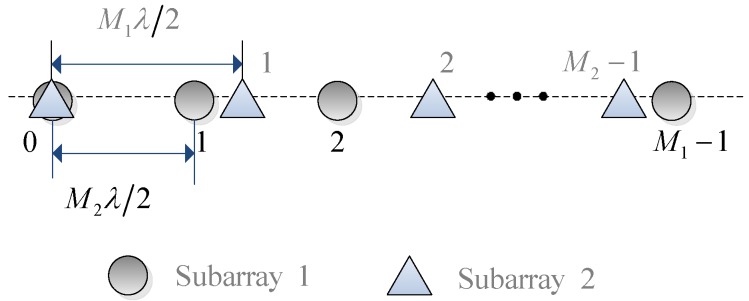
The system model of co-prime linear array.

As shown in Figure 1, we consider a co-prime linear array consisting of two uniform linear subarrays, which are located in the same line. The first subarray has $M_{1}$ equal-spaced omnidirectional sensors with inter-element spacing M2λλ22, while the second has $M_{2}$ equal-spaced omnidirectional sensors with inter-element spacing M1λλ22. Here $M_{1}$ and $M_{2}$ are co-prime integers, and *λ* is the wavelength. The two subarrays share the first sensor, and consequently the co-prime array has $M_{1}+M_{2}-1$ sensors.

Assume $K(K<\min\left(M_{1},M_{2}\right))$ uncorrelated narrowband signals impinge on the array. The received signal for the *i*th (*i* = 1, 2) subarray at time $t\left(1\leq t\leq T\right)$ is

(1) $$x_{i}\left(t\right)=\sum_{k=1}^{K}a_{i}\left(\theta_{k}\right)s_{k}\left(t\right)=A_{i}s\left(t\right)+n_{i}\left(t\right)$$

where $a_{i}\left(\theta_{k}\right)={\left[1,e^{-jM_{\tilde{i}}\pi \sin\left(\theta_{k}\right)}\ldots ,e^{-j\left(M_{i}-1\right)M_{\tilde{i}}\pi \sin\left(\theta_{k}\right)}\right]}^{T}$($i+\tilde{i}=3$) is the steering vector for the *k*th source $\theta_{k}$, $A_{i}=\left[a_{i}\left(\theta_{1}\right),\ldots ,a_{i}\left(\theta_{K}\right)\right]$ is the steering matrix, $s\left(t\right)={\left[s_{1}\left(t\right),\ldots ,s_{K}\left(t\right)\right]}^{T}$ is the source vector. The components of $n_{i}\left(t\right)$ are assumed to be independent and identically distributed additive white Gaussian noise with equal variance in each sensor, and are independent from the sources. ${\left(\cdot \right)}^{T}$ denotes the transpose operation.

According to the property of sinusoid function, for each source $\theta_{k}$ in the *i*th subarray, there exist some particular angles, denoted as $\theta_{i,k}^{eqv}$, that satisfy $\pi \sin\theta_{i,k}^{eqv}=M_{\tilde{i}}\pi \sin\theta_{k}+2n_{i}\pi$, where $n_{i}$ is an integer. From the definition of $a_{i}\left(\theta_{k}\right)$, the angle $\theta_{i,k}^{eqv}$ can be regarded as the equivalent angle for the true $\theta_{k}$ impinging on one traditional $M_{i}$-element ULA with half-wavelength spacing, which generates the same steering vector as $\theta_{k}$. The relation between true $\theta_{k}$ and its equivalent $\theta_{i,k}^{eqv}$ is thus given as

(2) $$\sin\theta_{i,k}^{eqv}=M_{\tilde{i}}\sin\theta_{k}+2n_{i}$$

**Figure 2:**
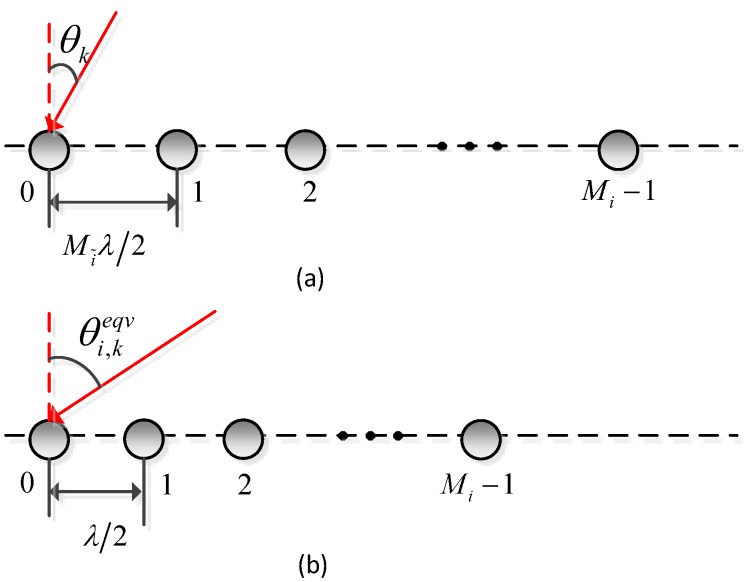
The System model of (**a**) the *i*th subarray and (**b**) its corresponding equivalent array of the co-prime linear array. $\theta_{k}$ is the true DOA for the *k*th source, and $\theta_{i,k}^{eqv}$ is the equivalent angle.

The signal model in Equation (1) amounts to *K* equivalent sources from angles $\theta_{i,k}^{eqv}$ impinging on a traditional ULA simultaneously. The system model of the *i*th subarray of the co-prime array and its equivalent uniform linear array is shown in Figure 2.

## 3. Proposed DOA Estimation Method

The equivalent DOA equals to the true one only when $M_{\tilde{i}}=1$. In the considered co-prime array, since $M_{\tilde{i}}>1$, there exist multiple equivalent angles $\theta_{i,k}^{eqv}$ for each true $\theta_{k}$. The true DOA $\theta_{k}$ may be one of the potential equivalent angles recovered by Equation (2), however, which one is the true DOA cannot be determined by each subarray alone. According to the property of coprimeness, the true DOAs can be uniquely estimated by finding the common angles in the two equivalent DOA sets generated by the two subarrays, respectively.

In the following, we aim to find the equivalent DOAs from Equation (1). To avoid the high complexity caused by spectral search, we propose to use the ESPRIT method.

### 3.1. Proposed Method

The steering matrix $A_{i}$ of the *i*th subarray can be rewritten as

(3) $$A_{i}=\left[\begin{array}{c} A_{i,1}\\ \mathrm{the}\;\mathrm{last}\;\mathrm{row} \end{array}\right]=\left[\begin{array}{c} \mathrm{the}\;\mathrm{first}\;\mathrm{row}\\ A_{i,2} \end{array}\right]$$

where $A_{i,1}$ is the first $M_{i}-1$ rows of $A_{i}$ and $A_{i,2}$ is constructed from the last $M_{i}-1$ rows. The relationship between $A_{i,1}$ and $A_{i,2}$ is

(4) $$A_{i,2}=A_{i,1}J$$

with $J=\mathrm{diag}\left(e^{-j\pi \sin\theta_{i,1}^{eqv}},e^{-j\pi \sin\theta_{i,2}^{eqv}},\cdots ,e^{-j\pi \sin\theta_{i,K}^{eqv}}\right)$.

The eigen-decomposition of the array covariance matrix $R_{i}=E\left[x_{i}\left(t\right)x_{i}^{H}\left(t\right)\right]$ of the *i*th subarray yields

(5) $$R_{i}=U_{s,i}\Lambda_{s}U_{s,i}^{H}+U_{n,i}\Lambda_{n}U_{n,i}^{H}$$

Here $E\left(\cdot \right)$ and ${\left(\cdot \right)}^{H}$ stand for statistical expectation and Hermitian transpose, respectively. $U_{s,i}$ and $U_{n,i}$ denote the signal- and noise-subspace eigenvectors, respectively. $\Lambda_{s}$ and $\Lambda_{n}$ contain the corresponding eigenvalues.

The signal subspace eigenvector $U_{s,i}$ can be similarly partitioned as

(6) $$U_{s,i}=\left[\begin{array}{c} U_{s,i,1}\\ \mathrm{the}\;\mathrm{last}\;\mathrm{row} \end{array}\right]=\left[\begin{array}{c} \mathrm{the}\;\mathrm{first}\;\mathrm{row}\\ U_{s,i,2} \end{array}\right]$$

where $U_{s,i,1}=A_{i,1}G$, $U_{s,i,2}=A_{i,2}G=A_{i,1}\mathrm{JG}$, and G is a full rank K×K matrix. Then, we have

(7) $$G^{-1}\mathrm{JG}={\left(U_{s,i,1}^{H}U_{s,i,1}\right)}^{-1}U_{s,i,1}^{H}U_{s,i,2}$$

The matrix ${\left(U_{s,i,1}^{H}U_{s,i,1}\right)}^{-1}U_{s,i,1}^{H}U_{s,i,2}$ has the same eigenvalues as J, while J contains the information of the equivalent DOAs. The equivalent DOAs can then be obtained by finding the eigenvalues of the matrix ${\left(U_{s,i,1}^{H}U_{s,i,1}\right)}^{-1}U_{s,i,1}^{H}U_{s,i,2}$. Specifically, assume the *K* eigenvalues are denoted as $d_{k}\left(k=1,2,\ldots ,K\right)$, the equivalent DOAs of the *i*th subarray can be estimated as

(8) θ^i,keqv=arcsindkdkππ

Let ${\hat{\theta }}_{i}^{eqv}=\left[{\hat{\theta }}_{i,1}^{eqv},{\hat{\theta }}_{i,2}^{eqv},\cdots ,{\hat{\theta }}_{i,K}^{eqv}\right]$. The candidate true DOAs can be recovered by Equation (2); i.e.,

(9) $${\hat{\theta }}_{i}^{cand}=\arcsin\left(\frac{1}{M_{\tilde{i}}}\left(\sin{\hat{\theta }}_{i}^{eqv}-2n_{i}\right)\right)$$

Here the relationship $-M_{\tilde{i}}\leq \left(\sin{\hat{\theta }}_{i}^{eqv}-2n_{i}\right)\leq M_{\tilde{i}}$ must hold due to the constraint of the sinusoid function. Hence, the set ${\hat{\theta }}_{i}^{cand}$ contains $M_{\tilde{i}}\times K$ candidate DOAs, among which only *K* angles are true. According to the coprimeness of $M_{1}$ and $M_{2}$, the true DOAs are obtained by finding the *K* common angles of the two candidate sets ${\hat{\theta }}_{1}^{cand}$ and ${\hat{\theta }}_{2}^{cand}$. In practice, due to the effect of noise, the true DOAs are estimated by seeking for the nearest angle pairs.

**Remark 1.**  *The two candidate sets ${\hat{\theta }}_{1}^{cand}$ and ${\hat{\theta }}_{2}^{cand}$ both contain the K true DOAs. In particular cases, except for true DOAs, they may have some other common angles (e.g., the candidate DOAs of different sources in different subarrays may have common angles). This may generate more than K common angles and cause ambiguity as a result. Without loss of generality, we consider K=2 sources, denoted as $\theta_{1}$ and $\theta_{2}$. Let $\theta_{c}$ both exist in the candidate set of source 1 in the i*th* subarray and the candidate set of source 2 in the $\tilde{i}\mathrm{th}$ subarray; i.e., except for $\theta_{1}$ and $\theta_{2}$, they generate another common angle. The relations are denoted as*

(10) $$\begin{array}{c} \left\{\begin{array}{c} \sin\theta_{c}=\sin\theta_{1}+\frac{2m_{\tilde{i}}}{M_{\tilde{i}}}\\ \sin\theta_{c}=\sin\theta_{2}+\frac{2m_{i}}{M_{i}} \end{array}\right. \end{array}$$

*where $m_{\tilde{i}}$ and $m_{i}$ are integers. Then we have the relation between $\theta_{1}$ and $\theta_{2}$ as*

(11) $$\sin\theta_{1}-\sin\theta_{2}=\frac{2m_{i}}{M_{i}}-\frac{2m_{\tilde{i}}}{M_{\tilde{i}}}$$

It is observed from Equation (11) that only when $m_{i}\neq 0$ and $m_{\tilde{i}}\neq 0$, the particular $\left(\theta_{1},\theta_{2}\right)$ pair will generate another common angle. For example, let us consider a co-prime linear array with $M_{1}=5$ and $M_{2}=7$ and assume $\theta_{1}=60.0^{{}^{\circ}}$ and $\theta_{2}=48.7^{{}^{\circ}}$. The two DOAs satisfy the relationship (11). The corresponding ambiguous DOAs generated by each subarray are listed in Table 1. As can be seen, for each source, the estimate of the two subarrays can generate one unique common angle—i.e., the true DOA. However, when the two sources ($\theta_{1}=60.0^{{}^{\circ}}$ and $\theta_{2}=48.7^{{}^{\circ}}$) are considered, expect for true DOAs, the estimate of the two subarrays may generate another two common angles (i.e., $-58.0^{{}^{\circ}}$ and $27.8^{{}^{\circ}}$). This violates the uniqueness and causes ambiguity as a result.

However, notice that the incident DOAs are in the continuous range between $-90^{{}^{\circ}}$ and $90^{{}^{\circ}}$, and only some particular angle pairs $\left(\theta_{1},\theta_{2}\right)$ that satisfy Equation (11) may generate another common ambiguous angle, except for the true DOAs. Specifically, for arbitrarily given $\theta_{1}$, only some discrete angles for $\theta_{2}$ follow the relationship Equation (11). Therefore, as compared to the continuous angle range, the number of $\left(\theta_{1},\theta_{2}\right)$ pairs that satisfy Equation (11) is rare. Therefore, the probability of the existence of a different common angle $\theta_{c}$ approaches zero and it is consequently a small probability event. Even if this small probability event happens, it can be solved by the entire co-prime linear array. The beamforming-related techniques, such as the classical beamforming approach (CBF) [18] and the Capon’s approach (also known as the minimum variance distortionless response, MVDR) [19,20], can be utilized to eliminate the problem. Specifically, the CBF spatial spectrum of the entire co-prime array is

(12) $$P_{CBF}=a_{C}^{H}\left(\theta \right)R_{C}a_{C}\left(\theta \right)$$

and the MVDR spatial spectrum is

(13) $$P_{MVDR}=\frac{1}{a_{C}^{H}\left(\theta \right)R_{C}^{-1}a_{C}\left(\theta \right)}$$

where $a_{C}\left(\theta \right)$ denotes the steering vector with respect to *θ* of the entire co-prime array, and $R_{C}$ is the covariance matrix of the entire co-prime array. By finding the peaks of $P_{CBF}$ in Equation (12) or $P_{MVDR}$ in Equation (13) the true DOAs can be estimated, and other ambiguous angles are eliminated. Thus, the ambiguity can be resolved successfully.

**Remark 2.**  *In general, there exist K common angles, which are the true DOAs. However, if the incident angles satisfy Equation (11), more than K common angles can be generated. We need to determine the number $\hat{K}$ ($\hat{K}\geq K$) of the common angles. Let $N_{1}$ and $N_{2}$, respectively, denote the numbers of the relevant angles (including the true and ambiguous angles) for the first and second subarray, and let $d_{i}$ denote the absolute value of angle difference, where $1\leq i\leq N_{1}N_{2}$. By sorting $d_{i}$ in ascendant order, we have*

(14) $$d_{1}\leq d_{2}\leq \cdots \leq d_{K}\leq \cdots \leq d_{N_{1}N_{2}}$$

*For common angles of the two subarrays, the angle differences are small (nearly 0), while they become larger for distinct angles. Define a decision variable as*

(15) $$D\left(n\right)=\frac{d_{n+1}-d_{n}}{d_{n}},n=K,K+1,\cdots ,N_{1}N_{2}-1$$

Notice that when both $d_{n}$ and $d_{n+1}$ are the differences of common angles or distinct angles, the decision variable will almost keep small. Meanwhile, if $d_{n}$ is the difference of common angles and $d_{n+1}$ is the difference of distinct angles, the decision variable will become much larger. Thus, the number of common angles can be estimated as

(16) $$\hat{K}=\arg\max_{n}D\left(n\right)$$

When $\hat{K}>K$, the true *K* DOAs can be distinguished according to Equations (12) and (13).

### 3.2. Complexity Analysis

The complexity of different methods is provided in Table 2. The ESPRIT method with *M* sensors and *K* sources requires covariance matrix estimation and the eigen-decomposition of both the covariance matrix $R_{i}$ and the matrix ${\left(U_{s,i,1}^{H}U_{s,i,1}\right)}^{-1}U_{s,i,1}^{H}U_{s,i,2}$, and the corresponding complexities are $O\left(M^{2}T\right)$, $O\left(M^{3}\right)$, and $O\left(3MK^{2}+2K^{3}\right)$, respectively. The resulting complexity of ESPRIT is given as $O\left(M^{2}T+M^{3}+3MK^{2}+2K^{3}\right)$. The complexity of the proposed method is given similarly. As is shown in Table 2, the complexity of the proposed method is lower than that of ESPRIT.

## 4. Results

In this section, we compare the proposed method with PSS [16] in co-prime arrays with $M_{1}=5$ and $M_{2}=7$. For fair comparison, the results of the traditional ESPRIT are also provided for two types of half-wavelength spacing ULAs with $M_{1}$ + $M_{2}$ − 1=11 and $M_{1}M_{2}$ − min$\left(M_{1},M_{2}\right)$ + 1=31 sensors, respectively. The former has the same sensor number as the considered co-prime array, but with less aperture length, denoted as ESPRIT with the same sensor number (ESPRIT-SSN). The latter has the same aperture length, but with more sensor elements, denoted as ESPRIT with the same aperture length (ESPRIT-SAL). The Cramer-Rao bound (CRB) for the co-prime array geometry is also given as a benchmark [21]. The searching grid for PSS is set as $0.1^{{}^{\circ}}$.

**Figure 3:**
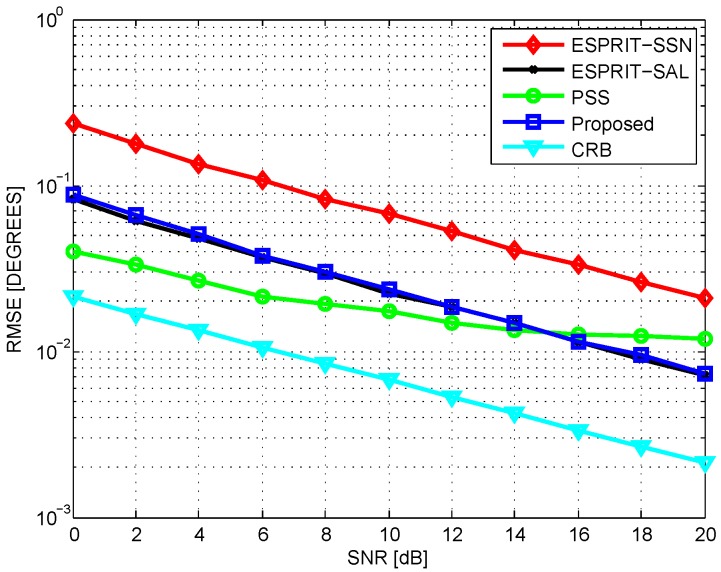
Root mean square error (RMSE) vs. signal-to-noise ratio (SNR), T=200. CRB: Cramer–Rao bound; ESPRIT: estimation of signal parameters via rotational invariance technique; ESPRIT-SAL: ESPRIT with the same aperture length; ESPRIT-SSN: ESPRIT with the same sensor number.

**Figure 4:**
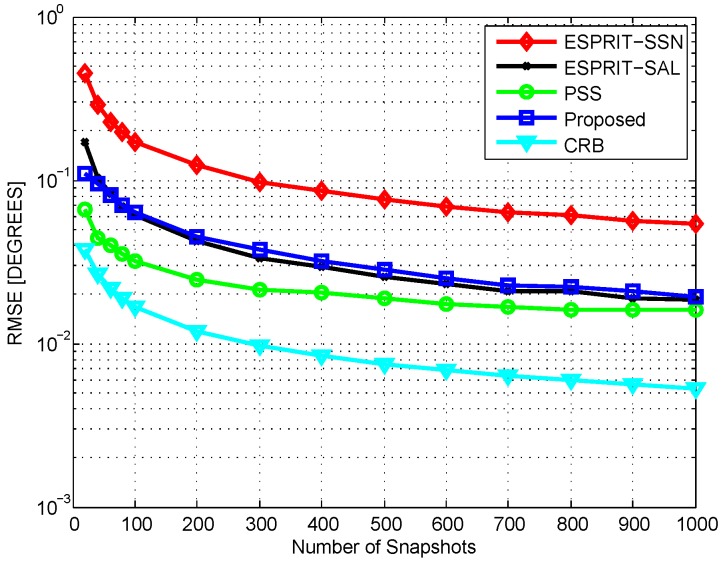
RMSE vs. Snapshot Number, SNR=5 dB.

In the first test, we compare the average root mean square error (RMSE) of different methods. Two uncorrelated sources are assumed to impinge on the array from directions $21^{{}^{\circ}}$ and $41^{{}^{\circ}}$. Figure 3 plots the RMSE performance versus signal-to-noise ratio (SNR) via 200 Monte Carlo simulations with T=200. As is shown, under the same sensor number condition, the proposed method outperforms ESPRIT-SSN greatly. With respect to the same aperture length, the proposed method can achieve almost the same estimation accuracy as ESPRIT-SAL. The proposed method is inferior to PSS at low SNR, and gradually exceeds PSS with the increase of SNR. Regarding the complexity, the ESPRIT-SSN, ESPRIT-SAL, PSS, and proposed method requires $O\left(2.57\times 10^{4}\right)$, $O\left(2.22\times 10^{5}\right)$, $O\left(3.17\times 10^{4}\right)$, and $O\left(1.55\times 10^{4}\right)$ complexities, respectively. The average running time for the four methods are 5.74 s, 15.60 s, 12.98 s, and 2.66 s. The complexity of the proposed method is lower than that of others, implying that the proposed method needs less memory. Especially, to achieve the same estimation accuracy, the proposed method only requires about 10% computational complexity as compared to ESPRIT-SAL. Therefore, the proposed method can achieve a better complexity–performance tradeoff. Further, Figure 4 depicts the RMSE performance versus snapshot number with SNR = 5 dB. The performance of the proposed method is almost the same as ESPRIT-SAL and is superior to ESPRIT-SSN, but is slightly weaker than that of PSS. However, the performance gap becomes smaller with the increase of snapshot number.

**Figure 5:**
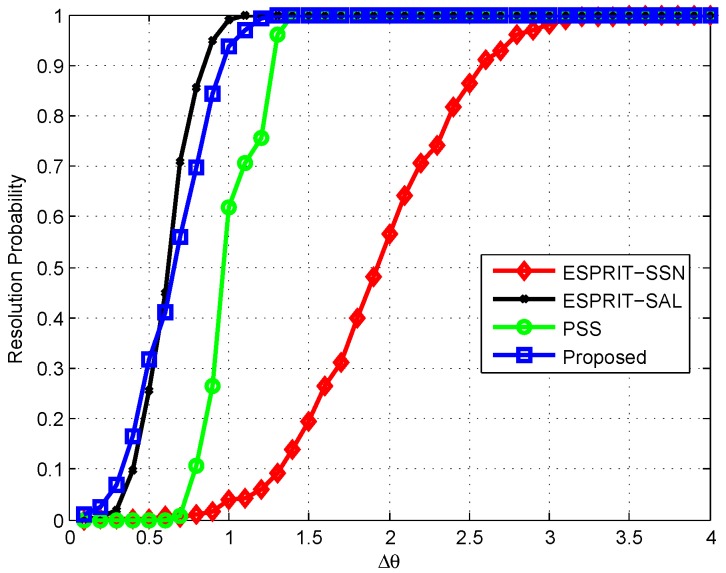
Resolution probability versus Δθ, with $\theta_{1}=20^{{}^{\circ}}$, $\theta_{2}=\theta_{1}+\Delta \theta$.

In the second test, we investigate the resolution probability performance. The two sources are said to be resolvable [22] if both $\left|{\hat{\theta }}_{1}-\theta_{1}\right|$ and $\left|{\hat{\theta }}_{2}-\theta_{2}\right|$ are smaller than θ1-θ2θ1-θ222, where $\theta_{k}$ and ${\hat{\theta }}_{k}$ (k=1,2) are the true and estimated DOAs, respectively. We set $\theta_{1}=20^{{}^{\circ}}$ and $\theta_{2}=\theta_{1}+\Delta \theta$, where Δθ is the control variable. We fix SNR=5 dB and T=200 and plot the resolution probability against Δθ in Figure 5. As can be seen, the resolution ability is enhanced with the increase of Δθ. The proposed method provides almost the same resolution performance as ESPRIT-SAL, but with substantially reduced complexity. The proposed method provides the best resolution performance as compared to ESPRIT-SSN and PSS.

To eliminate the ambiguity for some particular angle pairs that satisfy Eqaution (11), we give the ambiguity check results in Table 3. For $\theta_{1}=48.7^{{}^{\circ}}$ and $60.0^{{}^{\circ}}$ as in Table 1, the estimate of two subarrays may generate another two common angles except for true DOAs; i.e., $-58.0^{{}^{\circ}}$ and $27.8^{{}^{\circ}}$. We can see that the true DOAs are checked successfully by both CBF and MVDR. Notice that the spatial output powers for MVDR at true DOAs are much higher than that of other common DOAs, thus the MVDR-based check approach has superior resolution ability. However, since an inverse operation of the covariance matrix is required, the MVDR-based approach has a heavy computation burden. Therefore, the CBF-based approach is more feasible when real-time processing is required.

## 5. Conclusions

In this paper, we have proposed a low-complexity ESPRIT-based DOA estimation method for co-prime arrays. We firstly map the true DOAs into several equivalent angles for each source, which are regarded as the DOAs impinging on traditional uniform linear array. We obtain the equivalent DOAs by ESPRIT and then recover the candidate DOAs according to their relationship. Finally, we uniquely estimate the true DOAs by finding the common angles of the candidate DOAs of the two subarrays. Simulation results show that the proposed method achieves an improved complexity–performance tradeoff in terms of estimation accuracy and resolution ability, as compared to other existing methods.

## Figures and Tables

**Table 1:** Two directions of arrival (DOAs) and their corresponding ambiguous DOAs in each subarray.

$\theta_{1}=60.0^{{}^{\circ}}$	Subarray 1 with $M_{1}=5$	−***58.0***, −34.2, −16.1, 0.51, 17.1, 35.5, **60.0**
	Subarray 2 with $M_{2}=7$	−47.2, −19.5, 3.8, ***27.8***, **60.0**
$\theta_{2}=48.7^{{}^{\circ}}$	Subarray 1 with $M_{1}=5$	−74.4, −42.6, −23.1, −6.1, 10.4, ***27.8***, **48.7**
	Subarray 2 with $M_{2}=7$	***−58.0***, −26.7, −2.8, 20.6, **48.7**

**Table 2:** Computational complexity comparison.

ESPRIT	$O(M^{2}T+M^{3}+3MK^{2}+2K^{3})$
PSS [16]	$O(\left(M_{1}^{2}+M_{2}^{2}\right)T+M_{1}^{3}+M_{2}^{3}+\frac{J}{M_{2}}M_{1}\left(M_{1}-K\right)+\frac{J}{M_{1}}M_{2}\left(M_{2}-K\right))$
Proposed	$O(\left(M_{1}^{2}+M_{2}^{2}\right)T+M_{1}^{3}+M_{2}^{3}+3\left(M_{1}+M_{2}\right)K^{2}+4K^{3})$

Note: $M=M_{1}+M_{2}-1$, *J* is the number of sampling points. PSS: partial spectral search.

**Table 3:** Ambiguity check results of classical beamforming (CBF) and minimum variance distortionless response (MVDR) with T=200 and SNR=5 dB.

*θ*	$-58.0^{{}^{\circ}}$	$27.8^{{}^{\circ}}$	$48.7^{{}^{\circ}}$	$60.0^{{}^{\circ}}$
$P_{CBF}$|$P_{MVDR}$	*0.6122*|0.0931	*0.5974*|0.0926	***1.3082***|**0.9561**	***1.2529***|**0.9454**
True or false	*false*|false	*false*|false	*true*|true	*true*|true

## Abbreviations

DOA	Direction of arrival
ULA	Uniform linear array
ESPRIT	Estimation of signal parameters via rotational invariance technique
MUSIC	Multiple signal classification
TSS	Total spectral search
PSS	Partial spectral search
CBF	Classical beamforming
MVDR	Minimum variance distortionless response
ESPRIT-SSN	ESPRIT with the same sensor number
ESPRIT-SAL	ESPRIT with the same aperture length
CRB	Crame-Rao bound
RMSE	Root mean square error
SNR	Signal-to-noise ratio
